# Exploring the Potential of Dendritic Oligoglycerol Detergents for Protein Mass Spectrometry

**DOI:** 10.1007/s13361-018-2063-2

**Published:** 2018-10-01

**Authors:** Leonhard H. Urner, Yasmine B. Maier, Rainer Haag, Kevin Pagel

**Affiliations:** 1 Freie Universität Berlin, Institute of Chemistry and Biochemistry, Takustraße 3, 14195 Berlin, Germany; 2 Fritz Haber Institute of the Max Planck Society, Faradayweg 4-6, 14195 Berlin, Germany

**Keywords:** Oligoglycerol detergents, Native mass spectrometry, Tuning protein charge reduction, β-Lactoglobulin, Tandem mass spectrometry

## Abstract

The ability to design detergents that are suitable for protein analysis by mass spectrometry (MS) represents an on-going challenge in the field of native MS. Desirable detergent characteristics include charge-reducing properties and low gas-phase stabilities of complexes formed with proteins. In this work, the gas-phase properties of oligoglycerol detergents (OGDs) are optimized by fine tuning of their molecular structure. Furthermore, a tandem mass spectrometry (MS/MS) approach is presented that estimates the gas-phase properties of detergents simply by studying the dissociation behaviour of protein-detergent complexes (PDCs) formed with the soluble protein β-lactoglobulin (BLG).

**Electronic supplementary material:**

The online version of this article (10.1007/s13361-018-2063-2) contains supplementary material, which is available to authorized users.

**Graphical Abstract:**
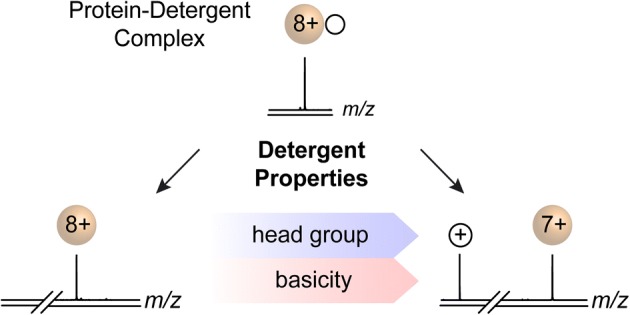
ᅟ

## Introduction

Detergents are commonly applied for the structural investigation of membrane proteins, which relies on the fact that they are used traditionally for membrane protein purification. A breakthrough in the field of proteomics came with the first demonstration that detergents can protect membrane protein complexes during the transport from solution into the vacuum of a mass spectrometer [1]. Membrane proteins that are encapsulated in detergent aggregates are usually transferred into the gas phase of a mass spectrometer by means of nanoelectrospray ionization (nESI), where the excess of detergent is subsequently removed via thermal activation [2, 3]. The increase in internal energy in complexes formed between detergent aggregates and membrane proteins mainly dissipates through the loss of detergent molecules, which thereby protects the protein structure from perturbation even under extremely harsh MS instrument conditions [4, 5]. The ability to gently transfer membrane protein complexes to the gas phase is useful not only for the elucidation of unknown subunit stoichiometry but also for assessing the structural impact of small non-covalently bound ligands, such as nucleotides, drugs, or lipids [6–10].

The activation conditions required for detergent removal and the charge state of the released protein are important parameters for the preservation of compact protein structures. High protein charge states together with high collisional activation promote Coulomb-driven unfolding of the protein, whereas low charge states in combination with soft release conditions are more likely to yield compact structures and to maintain native protein subunit stoichiometries [11]. Saccharide detergents, for example, are removed upon nESI at elevated activation energies and exhibit no charge-reducing properties, whereas detergents based on tetraethylene glycol or amine N-oxide, on the other hand, exhibit strong charge-reducing properties and are removed under gentle activation conditions [12]. The structural diversity of available detergent families makes it challenging to deduce design principles for detergents that allow for on-demand adjustment of gas-phase properties. To overcome this limitation, we here investigated the gas-phase properties of OGDs, an emerging class of non-ionic detergents that have not been yet considered for mass spectrometry applications.

Dendritic oligoglycerol is biocompatible, highly water-soluble and can be modified synthetically on large scales using established protocols [13–15]. The molecular architecture of OGDs is highly modular and comprises a dendritic oligoglycerol head group, a hydrophobic tail, and linker unit in-between. Earlier investigations focused mainly on the structure-based understanding of OGDs’ self-assembly in aqueous solution and the potential of their aggregates to be used as nanotransporters for hydrophobic drugs [16]. Here, we take advantage of their modular construction to improve the detergent’s propensity for protein charge reduction, which is highly desired for protein analysis by native MS.

**Scheme 1:**
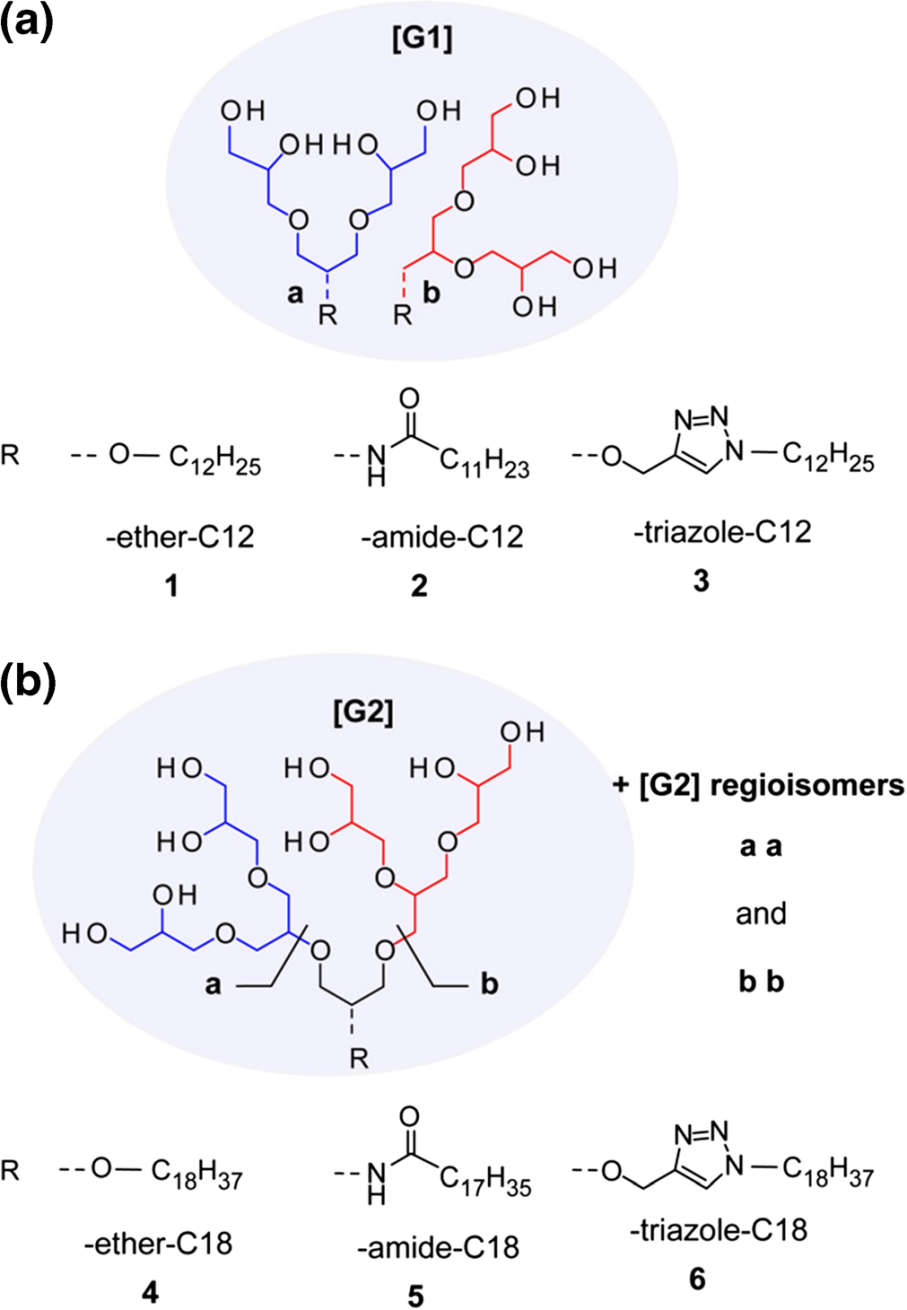
Overview of a) [G1]- and b) [G2]-detergent mixtures **1**–**6** to be explored for the investigation of protein-detergent complexes (PDCs) in the gas phase. The [G1]- and [G2]-detergents comprise similar regioisomer ratios, head groups and hydrophobic tails, but different linkages in-between (ether-, amide-, or triazole)

For this purpose, a small set of detergent batches was prepared, consisting of regioisomer mixtures derived from first- and second-generation oligoglycerol dendron [G1]–[G2], different linker groups (ether, amide, and triazole), and two different alkyl spacers (C12 and C18, Scheme 1). The structural diversity of **1**–**6** relies mainly on the size of their dendritic oligoglycerol head groups and the chemical nature of the linker that is embedded between the head group and the hydrophobic tail. The rationale behind the library design relies on the assumption that electrostatic and dipole-dipole interactions contribute most significantly to non-covalent interactions in the gas phase [17].

To evaluate the impact of individual detergent building blocks on the gas-phase properties of dendritic OGDs, three soluble proteins were assessed as model systems for the investigation of PDCs: two hydrophilic proteins, ubiquitin (8.5 kDa), and myoglobin (16.9 kDa) which are known to not bind detergents in solution under native conditions, and β-lactoglobulin (BLG, 18.4 kDa), a more amphiphilic protein that is suggested to play an important role in the transport of amphiphilic molecules [18]. In line with this suggestion, we found that BLG exhibits a greater propensity to form PDCs upon nESI than ubiquitin or myoglobin. Systematic tandem MS (MS/MS) experiments on singly bound PDCs formed between BLG and **1**–**6** revealed how distinct building blocks or functional groups of detergents mediate the removal of sodium ions and protons from protein ions during PDC dissociation.

## Experimental

### Materials and Reagents

All chemicals, solvents, and buffers were purchased from Merck (Germany), Sigma-Aldrich (Germany), Alfa Aesar (Germany), and Fluka (Germany). The OGDs **1**–**6** were synthesized using previously reported procedures [13–15].

### Preparation of Protein Detergent Mixtures

Proteins were dissolved in ammonium acetate (10 mM) buffer to a final concentration of 1 mg/mL. The protein samples (500 μL) were then loaded into Amicon-Ultra 0.5-mL centrifugal devices (Merck Millipore, Germany). The molecular weight cut-off (MWCOs) of the centrifugal devices was adjusted to the molecular weight of ubiquitin (MWCO = 3 kDa), myoglobin, and BLG (MWCO = 10 kDa). The samples were concentrated (14,000 ×*g*, 10 min) using a Heraeus Pico 17 centrifuge (Thermo-Scientific, USA), diluted with ammonium acetate buffer (10 mM) to a final volume of 500 μL, and concentrated again. This procedure was repeated five times. The protein concentration was determined upon the final centrifugation step by means of UV/VIS spectroscopy using the molar extinction coefficients at 280 nm of ubiquitin (1490 M^−1^ cm^−1^), myoglobin (holo form) (13,940 M^−1^ cm^−1^), and BLG (17,600 M^−1^ cm^−1^) [19–21]. Equimolar protein detergent mixtures (1:1, 50 μM) were prepared by appropriate dilution to generate a 1:1 complex stoichiometry, and the samples were subjected to nESI-MS analysis. The sample conditions, including protein concentration, buffer concentration, and concentration of the amphiphilic molecule, were adapted from Seo et al. [22].

### Nanoelectrospray Ionization Mass Spectrometry

Mass spectra were measured in positive ion mode using a modified Ultima high-mass quadrupole-time of flight (Q-ToF) mass spectrometer (Waters Micromass, Manchester, UK) equipped with a Z-spray nanoflow ESI (nESI) ionization source [23]. The required borosilicate capillaries were prepared according to a previously described procedure [24]. Data acquisition and analysis were performed by means of MassLynx (V4.1). Capillary voltage (1.2 kV), cone voltage (35 V), RF lens (50 V), collision gas pressure (P_argon_ ~ 5 · 10^−3^ mbar), and collision voltage (2 V) were adjusted to optimize the intensity of singly bound PDCs in the *m*/*z* range between 200 and 5000. The sample conditions and instrumental parameters are summarized in Scheme S1. External *m*/*z* calibration was performed by means of CsI solutions (20 mg/mL, H_2_O:isopropanol, *v*:*v*, 1:1). The intensities obtained from the individual protein signals (apo form) were extracted from the mass spectra and plotted as a function of the protein charge state. The data were fitted to a Gaussian function and the charge state (x-value) at maximum intensity was takes as the average charge state (*z*_ave_).

### Tandem Mass Spectrometry Experiments

For tandem mass spectrometry (MS/MS) experiments, the ions were selected according to their *m*/*z* using the quadrupole. The width of the isolated *m*/*z* channel was varied by using either high-resolution (LM res: 3.3 V, HM res: 15 V) or low-resolution settings (LM res: 3.3 V, HM res: 11.3 V). Ions of a specific *m*/*z* were subsequently activated by stepwise increasing the injection voltage into the collision cell (collision voltage). CID_50_ values were determined by plotting the disappearance of the precursor PDC ion population (*D*_PDC_) against the collision voltage:

$$ {D}_{\mathrm{PDC}}=\frac{I_{\mathrm{PDC}}}{I_{\mathrm{TIC}}}-{I}_{off} $$

To do so, the intensity of the PDC ion population (*I*_PDC_) was divided by the total ion count (*I*_TIC_). In case of an overlap in *m*/*z* with other ion populations that did not dissociate under the applied collision voltages, a constant offset (*I*_off_) was observed. This offset was subtracted from the individual *D*_PDC_ values. The resulting data points were fitted to a sigmoid function, the curves were normalized and the collision voltage at 50% intensity was taken as the CID_50_ value. Data analysis was performed manually by using Origin V9.1.

## Results and Discussion

### Mass Spectra of Protein Detergent Mixtures

**Figure 1:**
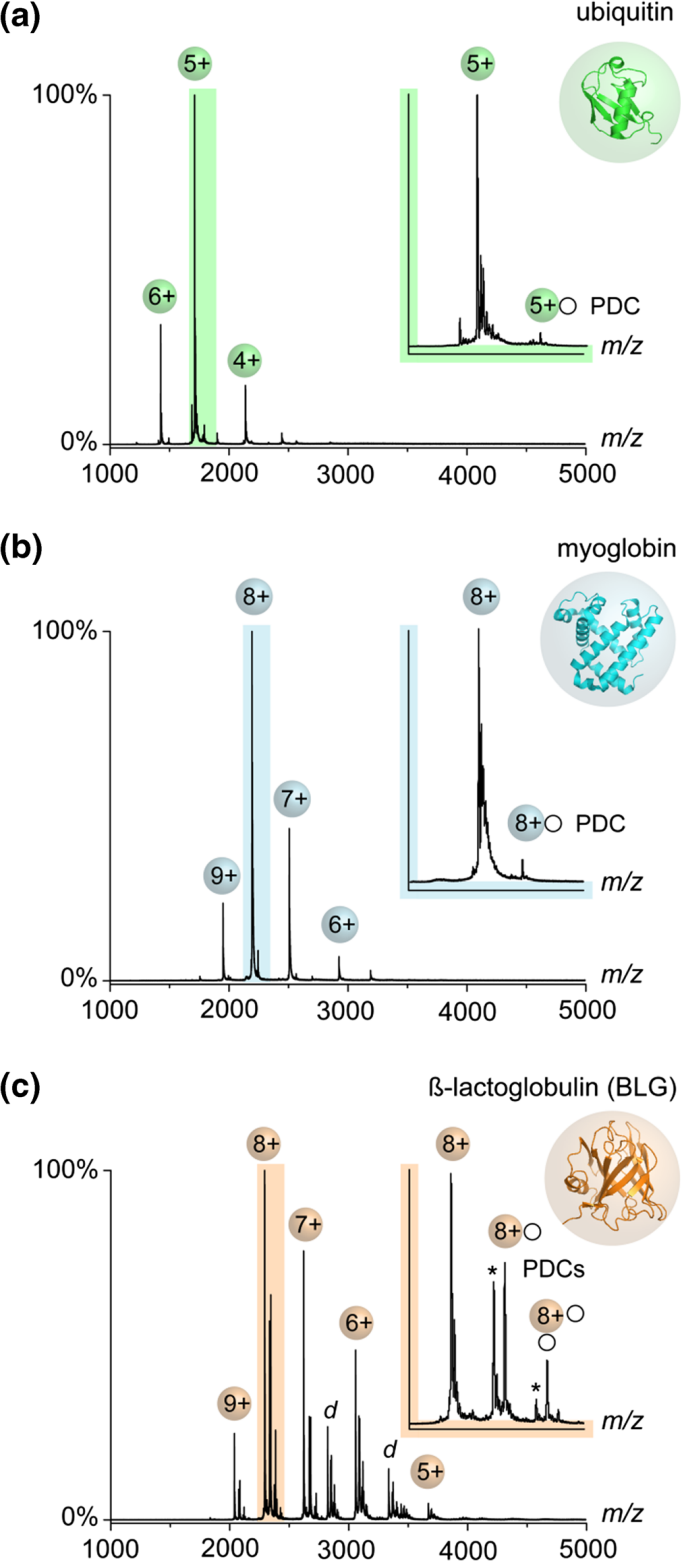
Mass spectra (nESI, positive mode) of three model proteins that were dissolved in NH_4_OAc buffer (10 mM) and in the presence of equimolar amounts of the detergent mixture **1**: (**a**) ubiquitin, (**b**) myoglobin (holo form), and (**c**) β-lactoglobulin (BLG). BLG dimers are labeled with *d* and lactosylated forms of BLG are labeled with an asterisk

In order to explore the impact of the detergent structure on the gas-phase properties of PDCs, it was necessary to identify a protein that shows sufficient binding to detergent molecules upon nESI. For this purpose, we analyzed equimolar mixtures of the non-ionic [G1] detergent mixture **1** with ubiquitin, myoglobin, and BLG. The mixtures were transferred into the gas phase by nESI from ammonium acetate buffer (10 mM) under instrumental conditions chosen to optimize the intensity of PDC signals (see supporting information). First, we tested ubiquitin and myoglobin. The mass spectra reveal low average charge states (*z*_ave_) for ubiquitin (5.0+) and myoglobin (8.0+, Figure 1a–b), which is commonly observed for both proteins under native MS conditions [22, 24]. Because only minor amounts of PDCs are observed, the mass spectra look remarkably similar to previously reported data from detergent-free protein samples [25, 26]. In contrast to the data obtained from ubiquitin and myoglobin, the mass spectrum of BLG shows three monomeric protein species that correspond to BLG bearing either no, one, or two covalently attached lactose moieties. For all monomeric forms of BLG, the formation of PDCs was obtained (Figure 1c) [27]. The *z*_ave_ of BLG (7.4+) on the other hand was equally low as in the case of ubiqutin and myoglobin [22].

For myoglobin and ubiquitin, no complex formation with non-ionic detergents in solution has been reported to date. Due to the high analyte concentration (50 μM), it is very likely that the obtained PDCs originate simply from non-specific adduct formation during the nESI process [28]. This implies that simply higher detergent concentrations are needed to maximize the PDC intensity, which is in line with previous observations [29, 30]. The outstanding propensity of BLG to show PDCs, on the other hand, is likely a result of its amphipilic properties. The barrel-like protein structure exhibits a hydrophobic core that is capable of forming inclusion complexes with amphiphilic molecules in solution [18, 31], which can be also transferred into the gas phase by nESI [32, 33]. To test in addition the capability of BLG to form PDCs via non-specific nESI contacts with detergents, we recorded mass spectra at higher detergent concentrations (> 50 μM, Figure S1). The intensity pattern observed among free BLG and its PDCs at high detergent concentrations can be perfectly described by a Poisson distribution, which clearly confirms that the PDC formation at high detergent concentration is triggered by enhancing the propability of randomly occurring contacts between protein and detergent molecules during nESI [12]. Considering the results for ubiquitin and myoglobin, it can therefore be concluded that at detergent concentrations of about 50 μM non-specific adduct formation during the nESI process contributes to the PDC intensity of BLG, but clearly only to a small extent (Figure 1c). Taken together, BLG was found to be the most suitable soluble protein for providing intense PDC signals under the experimental conditions employed.

### Dissociation Behaviour of PDCs—Impact of Ion Adducts and Detergent Structure

**Figure 2:**
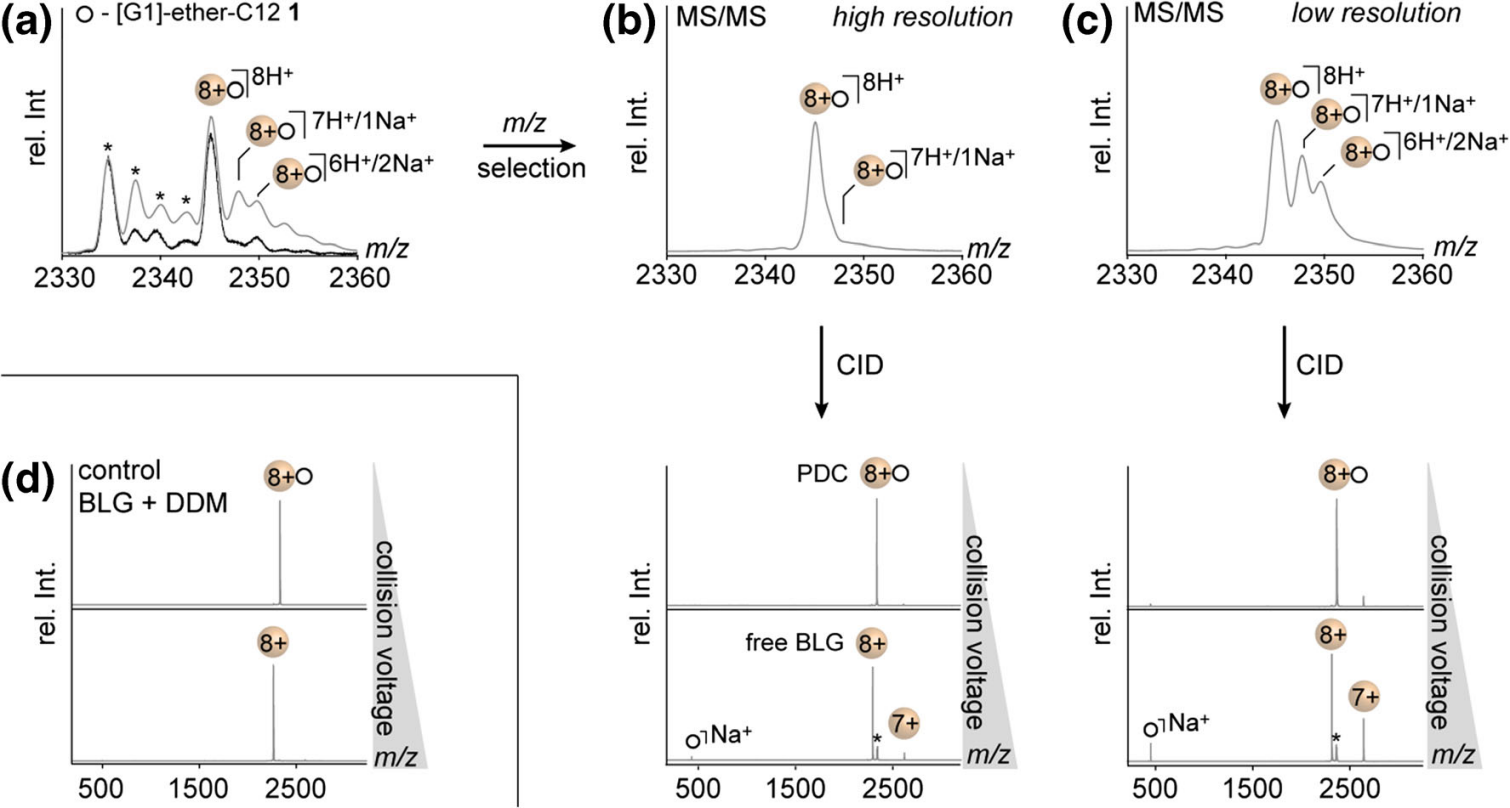
Tandem mass spectrometry (MS/MS) experiments reveal the influence of proton and sodium binding on the dissociation behaviour of PDCs formed by BLG and **1**. (**a**) Zoom into MS spectrum of the PDC at charge state (*z* = 8+) when analyzed in the absence (black) or presence of NaCl (500 μM, gray). (**b**–**c**) MS/MS spectra obtained from different quadrupole settings (top) and MS/MS spectra before and after collision induced dissociation (CID, collision voltage range: 2–30 V) of the parent ion populations. (**d**) For comparison, the results obtained for PDCs formed by BLG and DDM at low-resolution settings are shown. Lactosylated forms of BLG are labeled with an asterisk

Once BLG was identified as suitable model protein to obtain intense PDC signals upon nESI of equimolar protein-detergent mixtures, the dissociation behaviour of BLG PDCs was studied by means of MS/MS experiments. In order to take into account that the gas-phase dissociation of protein complexes is dependent on the protein charge states [34], the MS/MS experiments were focused systematically on the most abundant protein charge state 8+ (Figure 2). The mass spectrum of singly bound PDC ions obtained for BLG and **1** shows one intense peak for the fully protonated PDC and further signals of minor intensity that correspond to mixed sodiated forms (Figure 2). To examine the impact of sodium and proton binding, different quadrupole settings were used to enable the isolation of (i) mainly fully protonated- or (ii) mixtures of protonated and mixed sodiated PDC ions (Figure 2b, c, respectively).

To enhance the intensity of the mixed sodiated ions, sodium chloride (500 μM) was added to the samples (Figure 2). The selected ion populations were then thermally activated by increasing the collision voltage. Upon full dissociation of the PDC, the appearance of three new ion populations was observed corresponding to BLG of two different charge states (8+ and 7+) and singly sodiated detergent ions (Figure 2). Interestingly, the extent in BLG charge reduction is proportional to the amount of singly sodiated detergent ions that are released from the PDC (Figure 2b–c). This result shows that the charge reducing properties of **1** rely exclusively on the detergent’s ability to remove sodium cations from the PDC. Detergent molecules of **1** that do not capture a sodium cation are consequently dissociated from the complex as neutral species. Further experiments on the individual regioisomers of **1** revealed that the ability to remove sodium cations does not depend on the structure of the two trigylcerol head groups (Figures S2–3).

The origin of this behaviour possibly lies in the structural similarity between trigylcerol and 18-crown-6, which is known to have a high binding affinity to smaller alkali cations [35]. For comparison, we also tested the performance of the detergent *n*-dodecyl β-D-maltoside (DDM), which is an important standard in structural biology and one of the most widely used detergent applied in native MS. DDM and **1** exhibit similar alkyl spacers and linker groups, but differ in the structure of their head groups. The comparatively rigid disaccharide structure of DDM is less similar to that of crown ethers and as a consequence, no charge reduction of BLG is observed; irrespective of the sodium adduct intensity (Figure 2d). This implies that the triglycerol head groups of **1** are indeed the major coordination sites for sodium ions.

**Figure 3:**
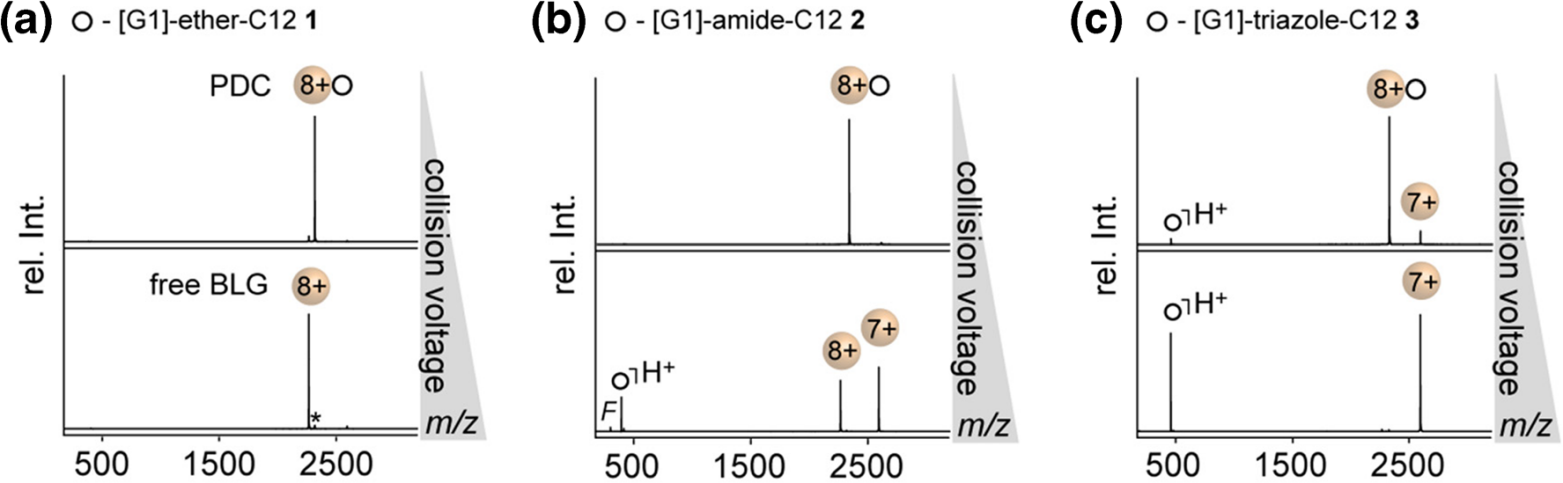
MS/MS spectra before and after full dissociation of PDCs (*z* = 8+, collision voltage range: 2–30 V) obtained from BLG and [G1] detergent mixtures **1**–**3**. High-resolution quadrupole settings were optimized to obtain maximum intensity of fully protonated PDCs prior to CID. Lactosylated forms of BLG are labeled with an asterisk; detergent fragments are labeled with *F*

To assess the impact of the linker between head group and tail, MS/MS experiments on fully protonated PDCs were performed (without the addition of sodium chloride) (Figure 3). In agreement with the results discussed above, no charge reduction of BLG is observed in the case of **1**, indicating that the detergent molecules dissociate as neutral species in the absence of sodium adducts (Figure 3a). Surprisingly, when exchanging the ether linkage between the detergent’s head group and tail for an amide bond **2** or triazole group **3**, an increase in protein charge reduction is observed (Figure 3b–c). The extent of protein charge reduction observed among **1**–**3** is proportional to the loss of protonated detergent molecules (Figure 3a–c).

Similar trends in charge reduction are observed for the [G2] detergent mixtures **4**–**6**, which gives clear evidence that the detergent’s ability to pick up a proton from the PDC is related to the chemical properties of the linker between head group and tail (Figure S4). This trend is expected because the affinity of the linkages to abstract a proton is directly correlated to their gas-phase basicity, e.g. ether (~ 790 kJ/mol), amide (~ 820 kJ/mol), and triazole group (~ 900 kJ/mol) [36]. In other words, the greater the basicity of the detergent, the more likely it is for a detergent to pick up a proton from the protein during PDC dissociation. This is also consistent with the difference in charge-reducing properties between DDM (790 kJ/mol) and lauryldimethyl-amine N-oxide (950 kJ/mol) observed during nESI-MS analysis of membrane proteins [12]. Taken together, the MS/MS data indicate that the dissociation behaviour is significantly influenced not only by the protonation/sodiation ratio of the PDC but also by the structural properties of the detergent, including the chemical properties of the head group and linker.

### Gas-Phase Stability of PDCs

The final objective of this study was to assess the impact of the detergent structure on the gas-phase stability of its PDC. To yield a descriptive value that allows a comparison of the stability of different PDCs, we determined the collision voltages required to dissociate 50% of the fully protonated PDC population during the CID experiment (CID_50_). The CID_50_ values were determined by plotting the depletion of the precursor ion population as a function of collision voltage. The data were normalized and fitted to a sigmoid function, and the collision voltage at 50% of the initial intensity was taken as the CID_50_ value (Figure S5).

Notably, the PDC formed by **4** is twice as stable as the PDC formed by **1** (Table 1). This trend is expected because the number of functional groups that contribute most likely to protein-detergent interactions increase by a factor of two, such as the ether backbone of oligoglycerol and the number of terminal hydroxyl groups (four vs eight).

**Table 1:** Overview of the charge reduction behaviour (*z* − 1) and stability (CID_50_) of PDCs (*z* = 8+) obtained from BLG and OGD mixtures **1**–**6** upon complete PDC dissociation (collision voltage ranges: 30–60 V)

OGD mixture		*z* − 1 (%)	CID_50_ (V)
**1**	[G1]-ether-C12	0	15
**2**	[G1]-amide-C12	56	13
**3**	[G1]-triazole-C12	100	11
**4**	[G2]-ether-C18	0	30
**5**	[G2]-amide-C18	43	25
**6**	[G2]-triazole-C18	100	13

Furthermore, by comparing the CID_50_ data among the [G1] series **1**–**3** and the [G2] series **4**–**6**, it becomes apparent that the extent of protein charge reduction is inversely correlated to the gas-phase stability of the PDC (Table 1). This leads to the conclusion that repulsive Coulomb interactions between the charged detergent molecules and the protein ions significantly lower the stability of the PDC. This conclusion is in line with the finding that the PDCs formed by **3** and **6** dissociate readily to a significant extent even at lowest collision voltages (Figure 3c). Taken together, the present data show that the stability of PDCs in the gas phase is governed by a balance between attractive dipole-dipole and repulsive Coulomb interactions. The stability of a PDC can be consequently up- or downregulated by tuning the number of functional groups that are involved in attractive dipole-dipole interactions. When the detergent picks up a charge from the PDC, however, repulsive Coulomb interactions are able to compensate attractive dipole-dipole interactions almost completely.

## Conclusion

In the present work, we evaluated the applicability of ubiquitin, myoglobin, BLG, and a set of six tailored dendritic OGD mixtures **1**–**6** for the investigation of PDCs in the gas phase of a MS instrument. Due to its outstanding propensity to show intense PDC signals upon nESI of protein-detergent mixtures, the soluble protein BLG was found to be most suitable for evaluating the gas-phase properties of detergents under the employed experimental conditions. MS/MS experiments on PDCs formed by BLG and **1**–**6** revealed that charge reducing properties of OGDs can be tailored by adjusting the intensity of sodium adducts or by fine-tuning the basicity of the detergent structure. The question left open is, if the results obtained from the PDCs formed by the soluble protein BLG and OGDs can be extrapolated to membrane proteins. It is known that charge reducing properties of detergents are not solely limited to membrane proteins; they can be also applied for charge reduction of soluble proteins, as shown by Reading and Liko et al. for the soluble protein transthyretin [12]. We therefore believe that our tandem MS approach based on the soluble protein BLG answers the principal question, if a detergent exhibits charge reducing properties or not. The data obtained from BLG and **1**–**6** furthermore clearly show how the detergents ability to remove charges from protein ions can be adjusted on-demand by varying the structure of distinct detergent building blocks or functional groups. Our findings therefore help to deduce design principles for the development of detergents with tailor-made gas-phase properties for native MS applications.

## Electronic Supplementary Material

ESM 1 (DOCX 960 kb)
