# Supplementary material for: Exploring the Potential of Dendritic Oligoglycerol Detergents for Protein Mass Spectrometry
Source: J Am Soc Mass Spectrom. 2018 Oct 1;30(1):174–80. doi: 10.1007/s13361-018-2063-2 (PMC6318253; doi:10.1007/s13361-018-2063-2)
Supplement: Supplementary file 1 — (DOCX 960 kb) [file 13361_2018_2063_MOESM1_ESM.docx]

**Supporting Information**


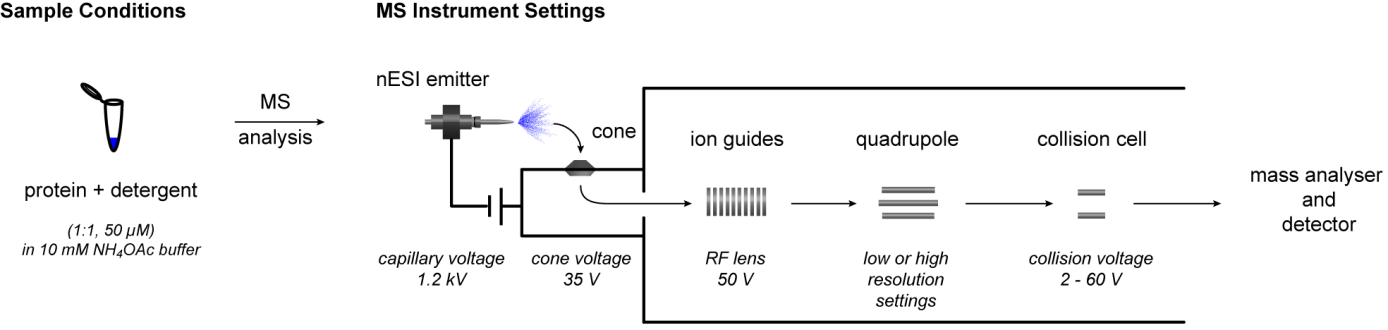


**Scheme S1.** Sample conditions and instrument settings applied for the investigation of PDCs in the gas phase. Argon was used as collision gas (5x10^-3^ mbar).

**
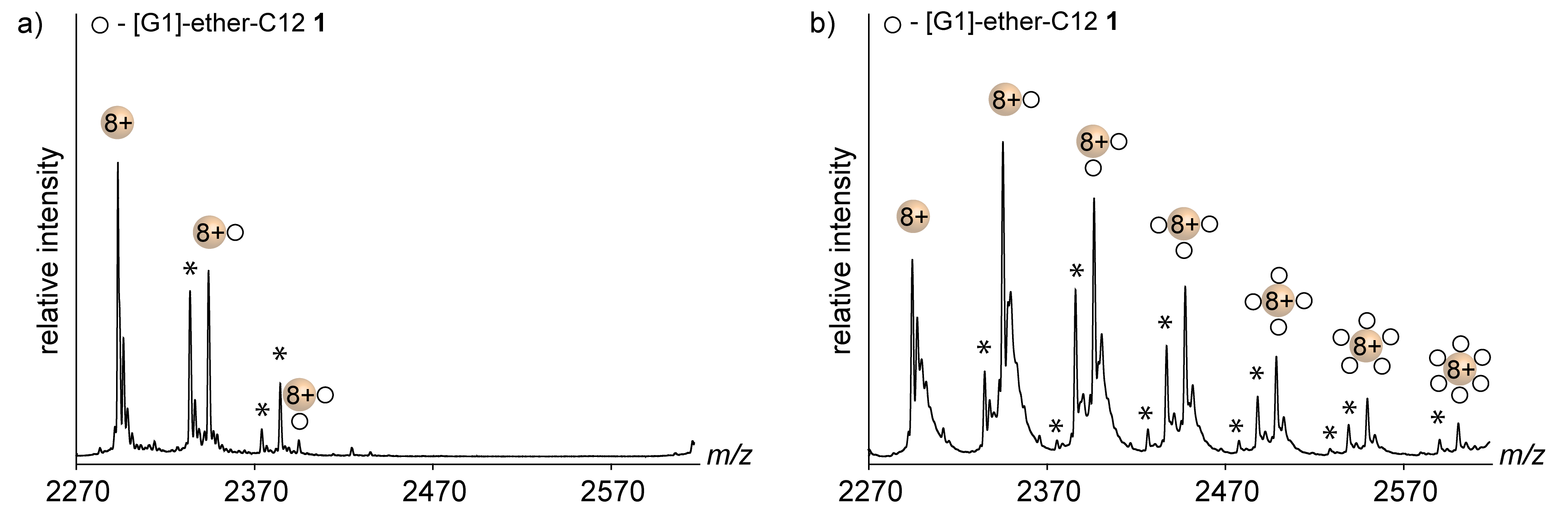
**

**Figure S1.** Zoom into MS spectrum of BLG at charge state 8+ is shown. The PDCs were obtained from mixtures between BLG and OGD **1**, which were analysed by nESI-MS using different detergent concentrations: a) 50 µM and b) 500 µM. The intensity profile among free BLG and its PDCs obtained from mixture b) can be described by a Poisson distribution, which indicates that non-specific contacts between BLG and OGD **1** during the nESI process contribute significantly to the formation of PDCs at high detergent concentration. Lactosylated forms of BLG and PDCs formed with lactosylated forms are labelled with an asterisk.

**
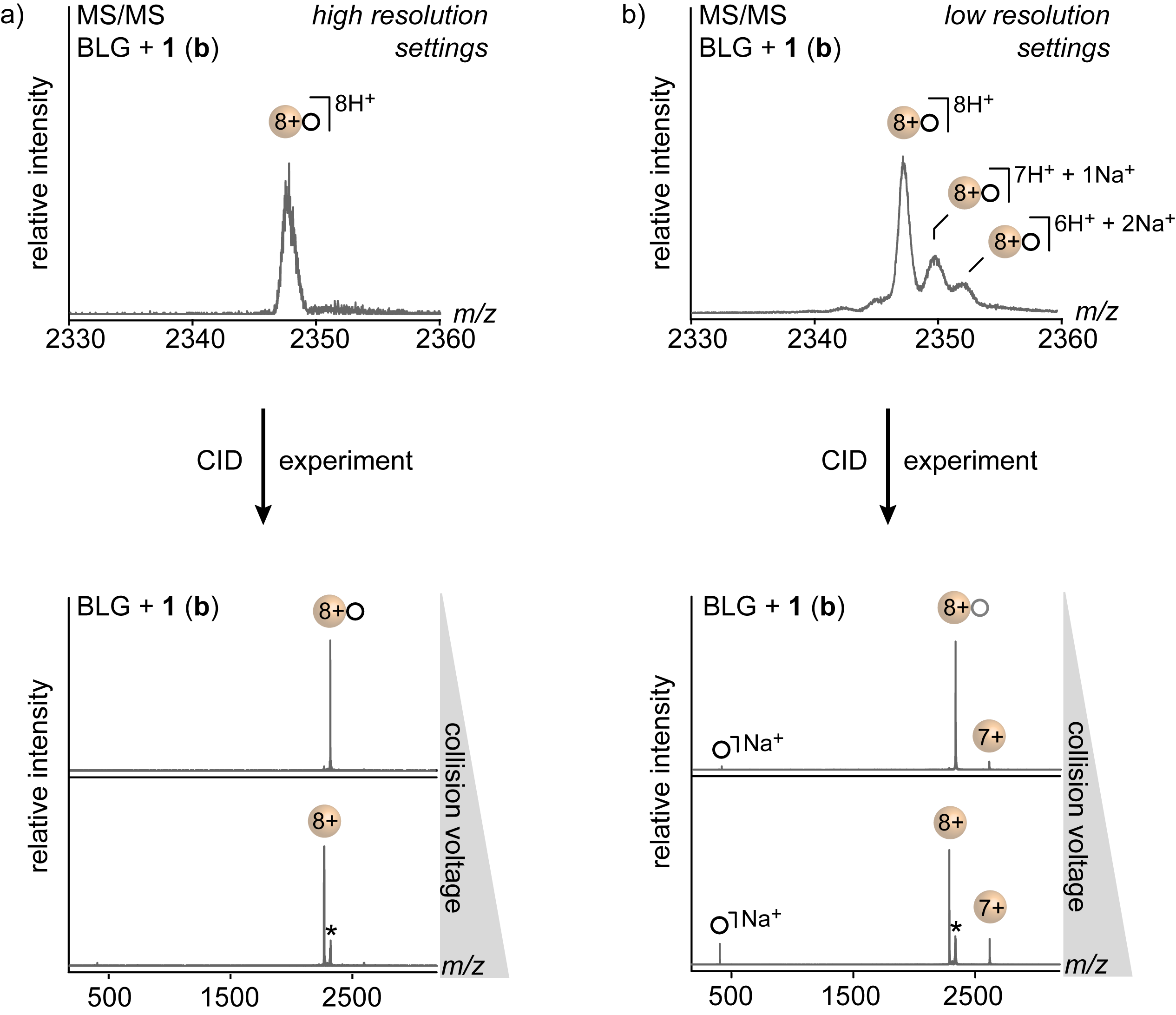
**

**Figure S2.** MS/MS experiments on PDC ions formed by BLG and **1** (**a**). a) Zoom into PDC spectrum at charge state (*z* = 8+) upon *m/z* selection with a) high- or b) low resolution quadrupole settings (top) and MS/MS spectra before and after complete dissociation of the selected parent ion populations (bottom, collision voltage range: 2 - 30 V). Samples were analysed by nESI-MS from ammonium acetate buffer (10 mM) and sodium chloride (500 µM).

**
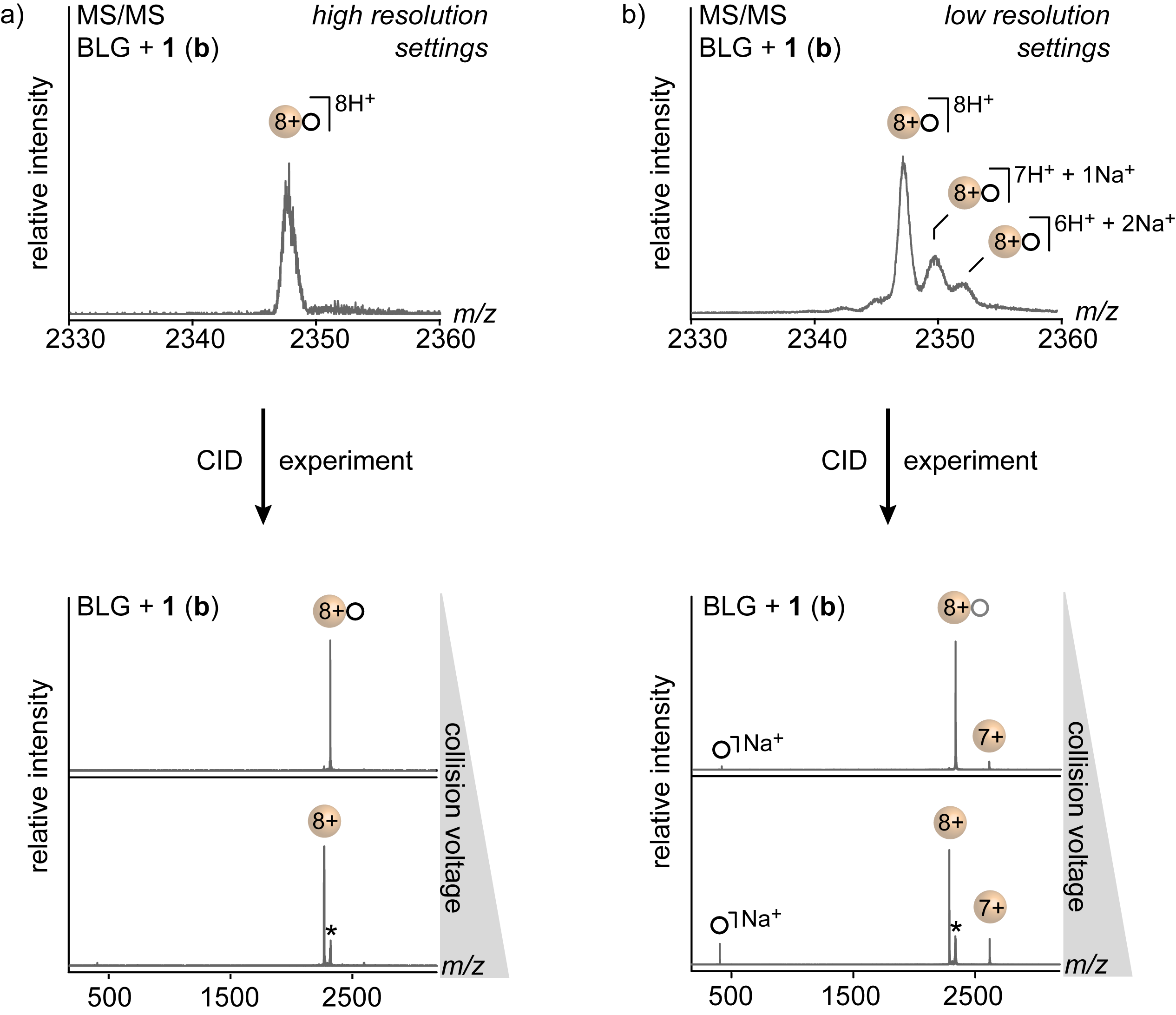
**

**Figure S3.** MS/MS experiments on PDC ions formed by BLG and **1** (**b**). a) Zoom into PDC spectrum at charge state (*z* = 8+) upon *m/z* selection with a) high- or b) low resolution quadrupole settings (top) and MS/MS spectra before and after complete dissociation of the selected parent ion populations (bottom, collision voltage range: 2 - 30 V). Samples were analysed by nESI-MS from ammonium acetate buffer (10 mM) and sodium chloride (500 µM).

**
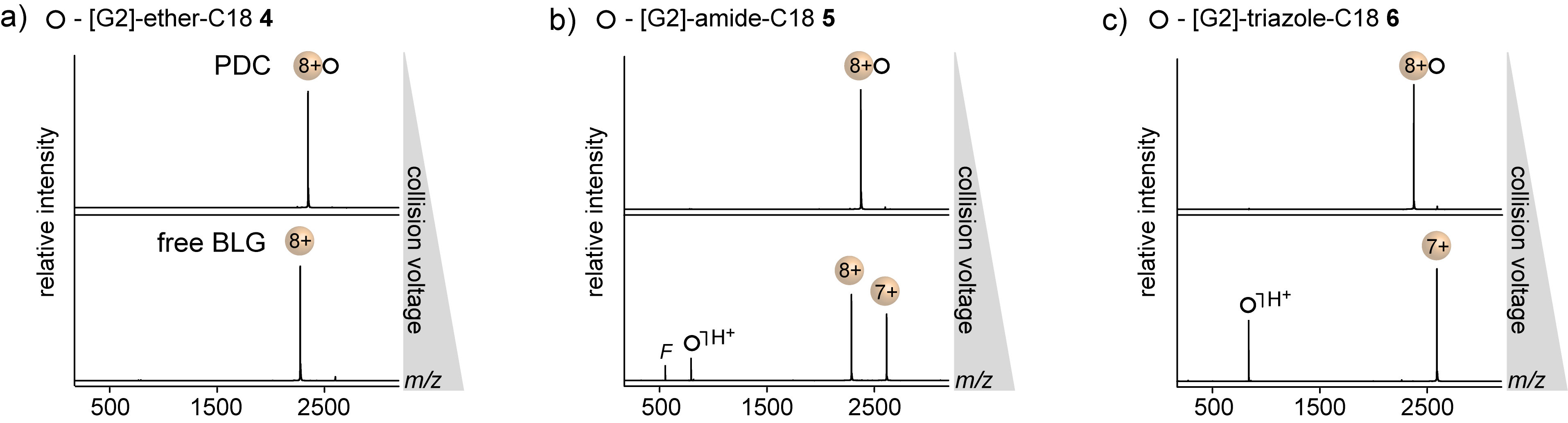
**

**Figure S4.** MS/MS spectra before and after full dissociation of PDCs (*z* = 8+) that were obtained from BLG and [G2] detergent batches **4** - **6** (collision voltage range: 2 - 60 V). High resolution quadrupole settings were optimized to obtain maximum intensity of fully protonated PDCs prior to CID. Lactosylated forms of BLG are labelled with an asterisk and detergent fragments are labelled with *F*.


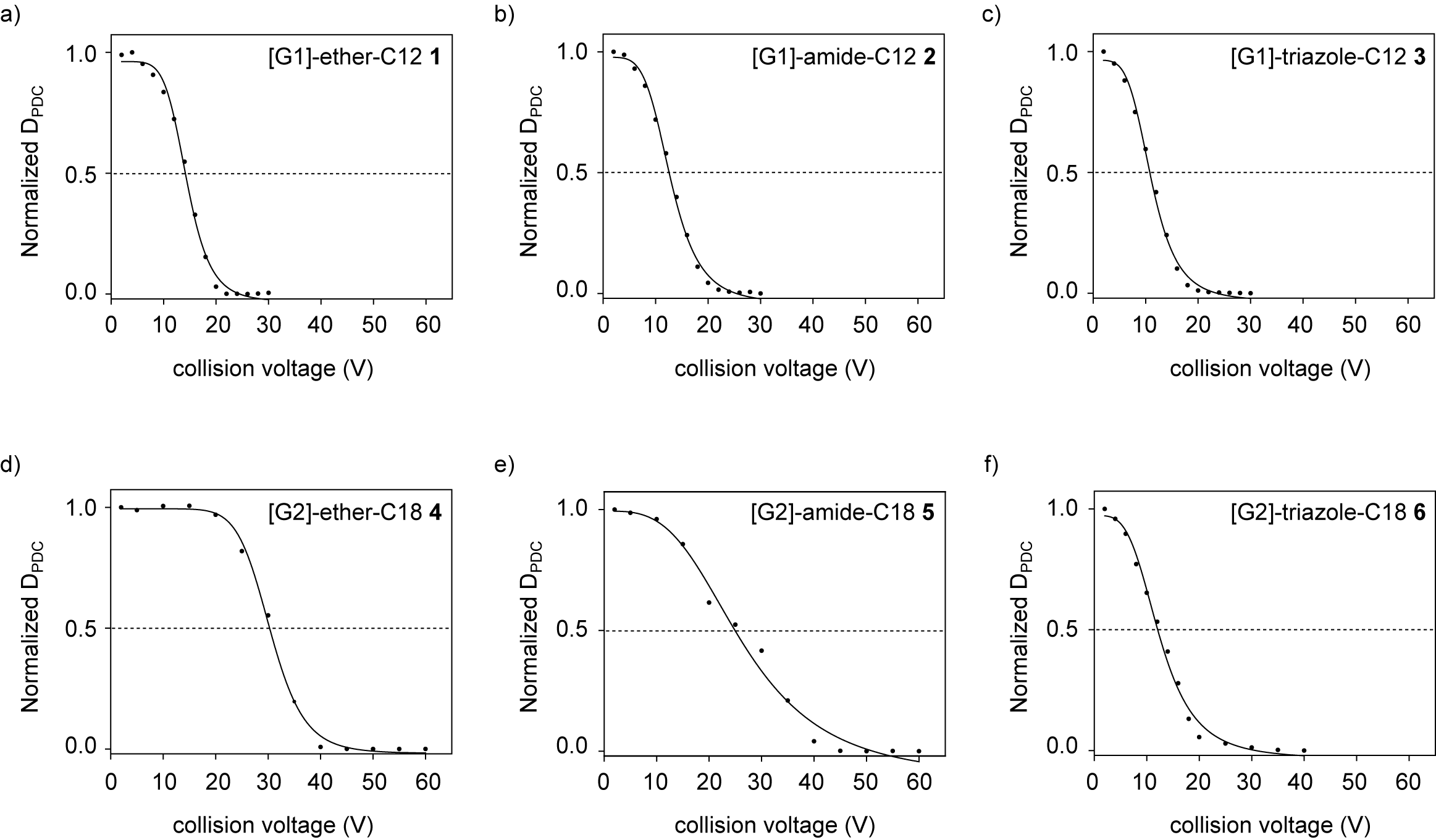


**Figure S5.** Normalized depletion of the parent PDC ion population (D_PDC_) against the collision voltage obtained for different detergent batches **1** ‑**6**. The collision voltage at 50% of the initial intensity was taken as the CID_50_ value.
